# Injectable Hydrogels for Cardiac Tissue Repair after Myocardial Infarction

**DOI:** 10.1002/advs.201500122

**Published:** 2015-07-15

**Authors:** Anwarul Hasan, Ahmad Khattab, Mohammad Ariful Islam, Khaled Abou Hweij, Joya Zeitouny, Renae Waters, Malek Sayegh, Md Monowar Hossain, Arghya Paul

**Affiliations:** ^1^ Center for Biomedical EngineeringDepartment of MedicineBrigham and Women's HospitalHarvard Medical SchoolCambridgeMA02139USA; ^2^ Harvard‐MIT Division of Health Sciences and TechnologyMassachusetts Institute of TechnologyCambridgeMA02139USA; ^3^ Biomedical Engineering and Department of Mechanical EngineeringFaculty of Engineering and ArchitectureAmerican University of BeirutBeirut1107 2020Lebanon; ^4^ Department of Electrical and Computer EngineeringFaculty of Engineering and ArchitectureAmerican University of BeirutBeirut1107 2020Lebanon; ^5^ Laboratory of Nanomedicine and BiomaterialsDepartment of Anesthesiology Brigham and Women's HospitalHarvard Medical SchoolBostonMA02115USA; ^6^ Laboratory for Nanoengineering and Drug DeliveryBrigham and Women's HospitalHarvard Medical SchoolBostonMA02115USA; ^7^ Department of Mechanical EngineeringFaculty of Engineering and ArchitectureAmerican University of BeirutBeirut1107 2020Lebanon; ^8^ BioIntel Research LaboratoryDepartment of Chemical and Petroleum EngineeringBioengineering Graduate ProgramSchool of EngineeringUniversity of KansasLawrenceKS66045USA; ^9^ Hamilton CollegeClintonNY13323USA; ^10^ Department of MedicineLyell McEwin HospitalUniversity of AdelaideSouth Australia5112Australia

**Keywords:** hydrogels, myocardial infarction, tissue engineering, regenerative medicine, cardiac repair, stem cell

## Abstract

Cardiac tissue damage due to myocardial infarction (MI) is one of the leading causes of mortality worldwide. The available treatments of MI include pharmaceutical therapy, medical device implants, and organ transplants, all of which have severe limitations including high invasiveness, scarcity of donor organs, thrombosis or stenosis of devices, immune rejection, and prolonged hospitalization time. Injectable hydrogels have emerged as a promising solution for in situ cardiac tissue repair in infarcted hearts after MI. In this review, an overview of various natural and synthetic hydrogels for potential application as injectable hydrogels in cardiac tissue repair and regeneration is presented. The review starts with brief discussions about the pathology of MI, its current clinical treatments and their limitations, and the emergence of injectable hydrogels as a potential solution for post MI cardiac regeneration. It then summarizes various hydrogels, their compositions, structures and properties for potential application in post MI cardiac repair, and recent advancements in the application of injectable hydrogels in treatment of MI. Finally, the current challenges associated with the clinical application of injectable hydrogels to MI and their potential solutions are discussed to help guide the future research on injectable hydrogels for translational therapeutic applications in regeneration of cardiac tissue after MI.

## 1 Introduction

Heart diseases are among the major causes of death worldwide. The main difficulties in the treatment of cardiac injuries arise from the limited capability of cardiac tissue to regenerate itself, i.e., the irreversibility of cardiac damages, the inability of cardiac tissues to tolerate ischemia, and the extremely limited period of viability of cardiac tissues postischemic injury.^1^, ^2^ Until now, the golden standard for treatment of cardiac damage has been heart transplantation. However, there is a huge gap between the large number of people who are approved for heart transplants and the small number of available donors, creating a severe shortage and thus an extreme limitation on the efficiency of this method alone. For example, for every 77 people who receive a heart transplant, 20 people die due to shortage while 98 000 remain on waiting lists of at least 3 years in the US alone.^3^ These statistics are even more grim in underdeveloped countries where the basic infrastructure required to achieve the timely linking of compatible donors to patients on waiting lists, and to perform the successful transplant itself, are lacking or nonexistent.

As a result, there is a huge need for developing new solutions to repair or replace damaged cardiac tissues. Injectable hydrogels have emerged as a promising approach for cardiac repair in regenerative medicine.^4^ The ability to rely on engineered or regenerated cardiac tissues, instead of a donated heart, would be a profound step forward in improving the rates of patient survival, as well as furthering the development of new treatments for myocardial infarction (MI), and many other life‐threatening maladies. Further importance of this approach arise from the fact that the body's natural response after MI is a process of fibrous remodeling that ultimately leads to scar formation instead of functional myocardium formation.^2^ This scar formation prevents the heart from functioning properly and eventually leads to complete heart failure. Injectable hydrogels have the potential to deliver therapeutic agents, cells or engineered tissues locally to the damaged area of the heart in order to regenerate functional cardiac tissue and, therefore, provide a revolutionary treatment option for MI. They provide an ideal and novel delivery strategy that has the ability to overcome the drawbacks associated with current treatments. In recent years, numerous injectable hydrogels have been developed and many of them have been tried for application in cardiac repair after MI. A number of reviews have also been published on the subject. However, to date, the clinical application of injectable hydrogels in repair or regeneration of post‐MI‐heart has not been realized.

In this article, we present a comprehensive overview of various injectable hydrogels for potential application in cardiac repair or regeneration. A brief overview of the pathology of MI has been presented in addition to the current clinical treatments and their limitations. The suitability of injectable hydrogels for cardiac regeneration has been discussed, along with other key important factors that need to be considered when designing the best hydrogel, the delivery method, and the therapeutics to be delivered. Finally, recent advancements in the field have been discussed followed by discussions on future perspectives for addressing the challenges hindering the widespread clinical application of injectable hydrogels in treatment of injured cardiac tissues.

## 2 Myocardial Infarction and Cardiac Tissue Damage

MI can be caused by a number of cardiac pathologies such as hypertension, blocked coronary arteries, and valvular heart diseases, thereby leading to ischemic cardiac injury.^2^ Death of cardiac cells, or myocardial necrosis, can occur as a result of insufficient blood flow which results in reduced supply of oxygen to the infarcted tissue.^5^, ^6^ Infarction disrupts the collagen fiber connections between cardiomyocytes and weakens the extracellular matrix which results in thinning and dilation of the ventricular wall. Abnormal stresses result in the healthy myocardium^7^ in an attempt to counterbalance the reduction in the cardiac function.^8^ This causes changes in the shape, structure, and functionalities of the heart and stimulates the body to respond to these changes and to remodel the injured ventricles^2^ leading to a cascade of consequences. Cytokines and growth factors are released to stimulate the immune systems in order to clean the infarcted region.^2^, ^5^ Fibroblasts, endothelial cells, and stem/progenitor cells at the infarcted zone each play important roles in the formation of granulation tissue, which is replaced by the extracellular matrix to form scar tissue. The newly generated scar tissue lacks the contractile properties needed for the heart to pump blood efficiently, which subsequently leads to heart failure.

## 3 Available Treatments to Repair Cardiac Damage after MI: Motivation for Use of Injectable Hydrogels

Three available treatments used for the remedy of cardiac tissue damage include pharmacological treatments, medical devices with interventional therapies including ventricular assist devices, and heart transplantation.^2^, ^9^ Medications are used to alleviate pain, reduce the cardiac workload and cardiac demand, and defend the heart from internally stored toxic substances. Medical devices and surgical therapies are used for various purposes such as restoring blood flow to the heart or reducing the generated stress with metallic and drug eluting stents or bypass auto grafting.^8^ These devices along with the interventional therapies optimize the functions of remaining viable cardiomyocytes, whereas transplantation replaces the damaged nonfunctioning tissues. The two techniques, namely, pharmacological treatments and medical devices with interventional therapies cannot sufficiently manage the progression of the disease and hence heart transplantation remains the only effective treatment^10^ which replaces the infarcted heart with a healthy heart from a donor.^11^ With an increasing number of patients, reduced number of donors and the immune complications that develop after heart replacement, transplantation remains an inadequate and inefficient technique.^10^, ^12^ Therefore, new methods are needed to tackle the issue more effectively. Cardiac repair using tissue engineering and regenerative medicine appears as a promising solution for cardiac repair after MI.^6^, ^13^

In both tissue engineering and regenerative medicine approaches, the repair of the cardiac tissue can be performed by transplantation of healthy living cells and/or suitable biomaterials into damaged cardiac environments. In regenerative medicine approach, the appropriate cell types are injected to the injured myocardium. These cells can be progenitor cells or stem cells that can be differentiated into the necessary cardiac cell types^5^, ^9^ or even fully differentiated cardiac cells. In one of the approaches of the regenerative medicine, some biomaterials‐alone can be injected near the infarcted heart tissue to provide the mechanical support to the injured heart tissue. In tissue engineering, the desired cell types are grown in some natural or synthetic biomaterials or sometimes even without any biomaterials, for the in vitro formation of cardiac tissue that could be implanted around the damaged areas of heart.^3^ By using these two techniques, different methodologies can be applied including cell delivery, cell‐encapsulated hydrogel delivery, hydrogels delivery with or without some biomolecules, and hydrogel delivery with both cells and biomolecules.^9^, ^14^, ^15^, ^16^, ^17^, ^18^

While in case of cell delivery without hydrogel, the low yield of cell retention at the targeted site is a concern, in the case of cell encapsulated hydrogel delivery, the hydrogel provides a matrix of support for the cells to adhere to prior to being injected into the body. Therefore, injectable hydrogels can overcome the problems of the lack of cell retention observed when cells have been injected by themselves and therefore they can produce more effective cardiac regeneration.^19^ In regards to delivering biomolecules or drugs, hydrogels can be used to encapsulate these substances, and be delivered locally to the targeted sites allowing to control the release kinetics and provide optimal sustained release of the molecules such as growth factors, chemokines and DNA plasmids that promote anti‐apoptosis, angiogenesis, and endogenous cell recruitment while preventing detrimental systemic effects.^7^

## 4 Injectable Hydrogels

Hydrogels are “water‐swollen polymer networks”^7^ that have a high percentage of water content identical to human tissues. They can be injected as a liquid and be crosslinked to a gel phase using certain physical or chemical stimuli. Alternatively, they can be injected in a partially crosslinked gel form as well. The gel formation after injection allows introducing the material inside the body in a minimally invasive way and permits the addition of bioactive molecules before the injection. Thermo‐sensitive hydrogels are specifically designed to initiate gelation at body temperature. Therefore, once injected, the transition from liquid to gel can occur.^20^, ^21^ Other methods of in situ cross‐linking can be photo cross‐linking, pH‐dependent crosslinking, or ionic crosslinking. Some hydrogels exhibit structures similar to that of the extracellular matrices, so once injected in the defected location, they can enhance the formation of a new extracellular matrix and improve integration within the body.^22^, ^23^ The design parameters during the synthesis of hydrogels play an important role in determining their properties and behavior. These design parameters need to be chosen based on the key properties that the hydrogel must exhibit and, therefore, must be ultimately based on the designated application of the hydrogel.

### 4.1 Design Parameters in Synthesis of Hydrogels

In order to design the optimal hydrogel, there are several parameters that need to be considered including the physical, material, and biological characteristics. In other words, the main factors that are essential to consider in the development of hydrogels in tissue engineering are the physical and material properties, primarily related to hydrogel mechanics, and the biological properties, involved in cell adhesion, for instance. Ideally, injectable hydrogels should be biocompatible, biodegradable, and bioresorbable to prevent triggering an immune response.^20^ The engineered tissue constructs, obtained using hydrogel, for cardiac implantation should be vigorous, contractible, and elastic with the ability to sustain periodic contraction and relaxation. They should also be well‐vascularized so that the cells encapsulated in them receive enough nutrients.^10^, ^24^ While natural polymers generally present more biocompatible features than synthetic polymers, the latter has greater varieties of structure, composition, and properties particularly better mechanical properties, which suggests a need for careful consideration of design parameters for the synthesis of hydrogels for cardiac applications.^25^, ^26^

While the cross‐linking density can be more easily controlled in covalently linked hydrogels, the formation of cross‐linking in ionic cross‐linking‐based hydrogel is controlled using multivalent counter‐ions.^27^ For example, the cross‐linking of hydrogels in situ with mild temperature fluctuation in polymerization is notably adapted in orthopedic applications.^28^ Maintaining an area for tissue growth, adhesion, and gene representation of cells is of paramount importance in the design of polymers. Scaffolds, cross‐linking types, density, and polymer‐chain rigidity are all important factors for consideration, while evaluating the mechanical properties of hydrogels.^29^ The design of the controlled degradation rate of hydrogels should take both hydrolysis and enzymatic reactions into consideration.^30^ Moreover, cell–hydrogel interaction contributes to adhesion which may be dependent on cell type, receptor–ligand interaction, and differentiation.^31^

### 4.2 Delivery Routes for Injectable Hydrogels

Along with investigating the proper hydrogels, cells, biomolecules, and drugs to promote the greatest amount of cardiac repair and regenerations, the optimal delivery pathway must also be established. When considering which pathway is the best, it is important to consider which methods produce the best results and are clinically viable. The main objective of any route of administration of therapeutics is to have optimal amount of active drugs delivered to the target site creating least risk to the patient. An important feature of injectable hydrogel is that it can be directly injected intramyocardially to the site of interest which potentially allows a minimally invasive treatment procedure with shorter hospitalization time.

Intramyocardial delivery involves injections of drugs or cells directly into the myocardium, usually into the left ventricle (LV) using an epicardial method or a catheter technique. While epicardial injections are reliable, this approach is invasive, limits access to the septum, risks puncturing the LV, and can lead to systemic embolization. The catheter‐based approach achieves better retention and avoids local toxicity effects but is also subject to the drawbacks of the epicardial method. Intravenous delivery is a low‐risk procedure and is minimally invasive but shows low cell retention and relies profoundly on cell homing.^32^ Intraperitoneal injections are tough to perform and injection sites are difficult to monitor.^33^ Disadvantages to nasal systemic drug delivery include variable amount of drug absorption, upper airway infections, and risk of long‐term damage to nasal epithelium.^34^

The use of locally injectable hydrogels has clear advantages over these other methods. First, the direct placement of material to damaged tissue makes the treatment pinpointed. Also, no surgery is needed using trans‐endocardial methods. Rather, electrophysiological guidance can be used to direct the injections. While controlling the amount of injected material is difficult and cell mortality during injection is inevitable, new strategies have been explored to mitigate these shortcomings including vacuum stabilization of the treated area.^35^ The feasibility of local injection methods coupled with the accurate and controlled delivery of therapies for damaged tissue makes this a desirable technique for MI treatment and requires further investigation.

### 4.3 Types and Compositions of Injectable Hydrogels

Injectable hydrogels can be made from a vast range of biomaterials that can be classified as either natural or synthetic in origin. Both natural and synthetic biomaterials present strengths and weaknesses in terms of biomedical applications that must be considered prior to the hydrogel synthesis. They can also be divided into two types depending on the type of cross‐linking: either a chemical cross linking type, linked by covalent bonds or a physical cross linking type, physically connected by combining polymeric chains and nanoparticles.^20^ Some of these hydrogels both natural and synthetic types are discussed below.

#### 4.3.1 Natural Polymer Based Hydrogels

**Figure 1:**
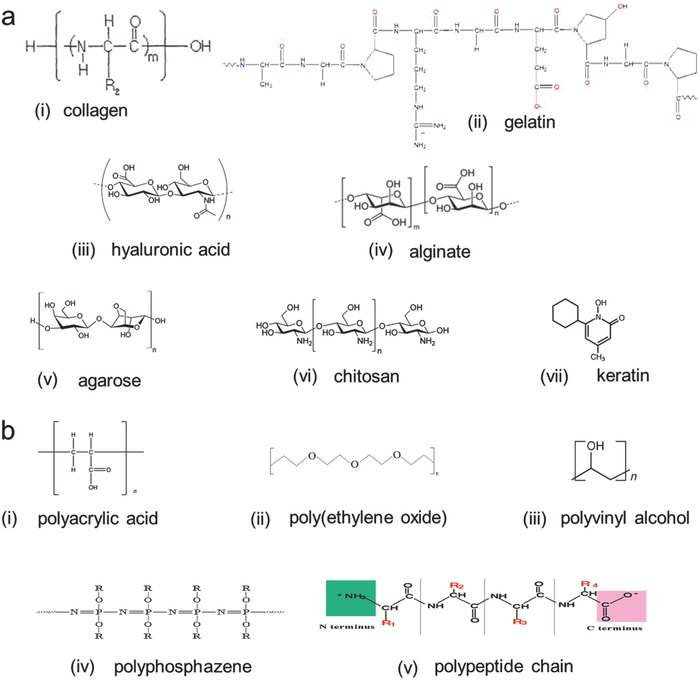
Chemical structures of various natural and synthetic hydrogels that have been tried or have the potential for application as injectable hydrogels in cardiac tissue regeneration. a) Some natural hydrogels: i) collagen,^134^ ii) gelatine,^135^ iii) hyaluronic acid,^135^ iv) alginate,^136^ v) agarose,^137^ vi) chitosan,^138^ and vii) keratin. b) Some synthetic hydrogels: i) polyacrylic acid, ii) poly(ethylene oxide), iii) polyvinyl alcohol, iv) polyphosphazene, v) polypeptide chains.

Naturally derived hydrogels are usually biocompatible and supportive of cellular activities. However, they have low mechanical strength, may induce an immune response, and are subject to batch‐to‐batch variations. Furthermore, structural modification is difficult due to their structural fragility and complexity,^36^] **Figure** **1**a. Despite these drawbacks, naturally derived biomaterials continue to be a promising component of hydrogels due to their bioactivity and the fact that many of them are naturally present in the human body. This correlation can be extremely valuable because one of the major goals of hydrogel synthesis is to produce tissues that are analogous to the native tissues. The characteristics of specific natural biomaterials are discussed below as well as how these characteristics pertain to injectable hydrogels.

*Collagen*: Collagen is a natural polymer; present in the extracellular matrix of skin, bone, tendons, ligaments, and cartilage of mammalian tissues, Figure 1a(i). It is composed of combinations of amino acid sequences that are biocompatible in terms of cell recognition and are amenable to enzymatic degradation in the presence of collagenases. The advantages of using collagen include its biocompatibility, strong cellular activities, and thermal reversibility. The disadvantages of using collagen for hydrogel synthesis include its low physical strength or inferior mechanical properties, high synthesis cost, and inherent immunogenic responses. In order to improve the physical properties of collagens, chemical cross‐linking using glutaraldehyde^37^ or diphenylphosphorylazide^38^ is recommended. Collagen has been widely used as a scaffold for 3D cell cultures and tissue engineering of artificial skin. The cell–collagen attachment mechanisms can be controlled through chemical modification by incorporating fibronectin, chondroitin sulfate, or low levels of hyaluronic acid into the collagen matrix.^39^ Collagen has been successfully used as a natural polymer based hydrogel to repair MI in vivo. Dai et al. investigated the use of collagen hydrogels (named Zyderm from INAMED Corp which consisted of 95% collagen type I and 5% of collagen type III) into rat MI model.^40^ Results showed improved stroke volume (SV), ejection fraction (EF), and wall thickness in collagen hydrogel group compared to the controls. The authors claimed that the high density and concentration of injected collagen provided the beneficial outcomes, suggesting that collagen hydrogels with proper optimization are a promising biomaterial for cardiac tissue repair.

*Gelatin*: Gelatin, Figure 1a(ii), is formed by decomposing the collagen triple‐helix structure into single strand molecules. Furthermore, the preparation of gelatin is distinguished by the postbreakage treatment of the collagen structure. Acidic treatment yields gelatin of type A, while alkaline treatment, characterized by high carboxylic content, yields gelatin of type B.^41^ Altering the solution temperature leads to the formation of gelatin that is renowned for its high biocompatibility and simplicity. Vascularization in tissue engineering is promoted through gelatin gels that present growth factors for delivery.^41^ Being a natural polymer and a derivative of collagen, gelatin also has a high potential for application in cardiac repair after MI.

*Hyaluronic Acid*: Hyaluronate, a component of glycosaminoglycan, is formed into hydrogels by covalent cross‐linking with hydrazine derivatives^42^ along with the radical polymerization of glycidyl methacrylate,^43^ Figure 1a(iii). Hyaluronidase, a primary enzyme found in cells and serum, plays roles in degradation of hyaluronate. The application of hyaluronate has been investigated in various tissue engineering applications such as wound healing and development of engineered skin tissues and intradermal implants.^44^ One major disadvantage of hyaluronate, similar to other natural hydrogels, is its weak mechanical properties that hinder the scope of its applications. However, the properties can be improved or controlled by modifying the molecular structure and composition with various functionalization. In a recent study, a methacrylated hyaluronic acid (MeHA) macromere crosslinked with tetramethylethylenediamine (TEMED) and ammonium persulfate (APS) was applied in an MI model to ligate the descending and diagonal coronary artery.^45^ The results showed significantly reduced infarct expansion and improved cardiac function. In another study, hyaluronic acid was crosslinked with PEG‐SH_2_ after acrylation and applied as an injectable hydrogel at epicardial surface of infarcted site.^46^ It was found that the modified hyaluronic acid hydrogel provided a significantly higher EF and SV index reduced infarct size, increased wall thickness, and better vessel formation compared to those of MI control group, suggesting that proper modification and optimization of hyaluronic acid hydrogels can offer promising solution for cardiac tissue repair after MI.

*Fibrin*: Fibrin gels are formed at ambient temperatures by polymerization of fibrinogen with thrombin as a crosslinking agent.^47^ Cell‐associated enzymatic activity promotes the degradation of fibrin during cell migration, the rate of which is controlled by apronitin, a proteinase inhibitor.^48^ Matrix synthesis along with cell migration and proliferation is promoted with the use of fibrin gels in a mechanism yielding the incorporation of platelet‐derived growth factors along with the transforming growth factor *I* (TGF‐*I*).^49^ Incorporation of the domain peptides (with a factor XIIIa substrate in one domain and a bioactive peptide containing RGD sequence in another domain) into fibrin gels, by the action of transglutaminase during the process of coagulation, constitutes the gateway for a variety of neurological applications of fibrin.^50^ In multi­ple studies, injection of fibrin into the infarcted area of rat left ventricle resulted in increased cell transplant and survival, decreased infarct size, increased blood flow to ischemic myocardium, and improved cardiac function.^51^, ^52^ Fibrin joins the ranks of hyaluronate with its mechanical properties limitations, reducing its scope of applications in the tissue‐engineering field. Natural wound healing and 3D scaffolds for tissue engineering are common applications of fibrin. Its properties in cell adhesion along with its capability to synthesize from the host's blood without triggering any inflammatory responses make fibrin highly attractive.

*Alginate*: Alginate, also known as alginic acid or algin, hydrogels are anionic polysaccharides, extracted from the cell walls of a brown algae. They are primarily used in drug delivery for wound healing, due to their high biocompatibility, low cost, and simple gelation with Ca^2+^, Mg^2+^, Ba^2+^, and Sr^2+^,^53^ Figure 1a(iv). The transplantation of chondrocytes, hepatocytes, and islets of Langerhans has been used with alginate gel beads.^54^ One main disadvantage of alginate hydrogel is its release of divalent ions to surrounding, resulting in limited long‐term stability. This mechanism can be counteracted with covalent cross‐linking using a variety of molecules which produce different cross‐linking densities.^30^ Another solution involves the isolation of polyguluronate blocks from alginate and subsequent oxidation and covalent cross‐linking of these derivatives with adipic acid dihydrazide.^30^ Oxidization of alginate leads to its degradation in aqueous media with temperature and pH being control factors.^55^ The hydrophilicity of alginate constitutes a disadvantage in terms of protein absorption. However, ligand‐specific binding properties can be enhanced by modifying with lectin or other ligands. When covalently coupled with an RGD containing cell adhesion ligand, the adhesion and proliferation of differentiated phenotypes of skeletal muscle cells were significantly enhanced.^56^ Alginate hydrogel has also been tested in cardiac applications for treatment after MI, due to its nonthrombogenic properties.^57^, ^58^ In another study, a calcium crosslinked alginate hydrogel was tested in rat MI model and was found to be significantly effective in reducing left ventricular (LV) expansion and increasing the old‐infarcted heart wall thickness.^59^ Similarly, alginate also showed promising results in large animal models such as a porcine model for treatment of MI.^60^ Consequently, alginate has become the very first injectable material to enter clinical trials for the treatment of MI (*Ikara Holdings, Inc. IK‐5001 for the Prevention of Remodeling of the Ventricle and Congestive Heart Failure After Acute Myocardial Infarction. NCT01226563; 2010*).

*Agarose*: Agarose, Figure 1a(v), like alginate, is a marine extracted algal polysaccharide except that it can form thermally reversible gels in a structure composed of double‐helices and junction zones consisting of multiple chain aggregations.^61^ Modifying the physical structure of agarose by controlling the concentration can alter the pore size of the gel. By combining the properties of large pores and minimal stiffness at low concentrations cell migration and proliferation can be enhanced.^61^ For an enhanced cell interaction mechanism, cell adhesion peptides (CDPGYIGSR) have also been covalently coupled with agarose.^62^ In a recent study, agarose was used for encapsulation of cardiac stem cells (CSCs) and their delivery to an infarcted heart which resulted in enhanced cardiac repair and blood vessel formation.^63^

*Chitosan*: Chitosan hydrogel is prepared by *N*‐deacetylation of chitin and is characterized by high biocompatibility and low toxicity due to its structural similarity to natural glycosaminoglycans, Figure 1a(vi). In addition, its solubility in an acidic environment, due to its high crystallinity, makes the application of chitosan highly effective. The hydrogel formation process of chitosan is performed by ionic^64^ crosslinking or chemical cross‐linking with glutaraldehyde. Chitosan modification with sugar residues^65^ and proteins such as collagen and albumin have yielded promising results in tissue‐engineering.^66^ Chitosan‐based hydrogels can be used to protect the transplanted cells, promote angiogenesis, reduce infarct size, and improve cardiac function due to their ability to conjugate with various bioactive molecules. This can increase their bioactive functions without affecting their physical/chemical properties, such as gelation behaviors, swelling and degradation characteristics, network structure, and mechanical strength.^67^ In a recent study, Lu et al. used chitosan with β‐glycerol phosphate and hydroxyethyl cellulose to synthesize a temperature‐sensitive hydrogel and applied in a rat MI model.^68^ A significant improvement was found in infarct size, wall thickness, microvessel, end systolic diameter (ESD), and end diastolic diameter (EDD). Two other independent studies also reported similar results for the thermo‐responsive chitosan hydrogel in rat MI model, demonstrating its high effectiveness in cardiac tissue repair.^69^, ^70^

*Keratin*: Keratin, Figure 1a(vii), represents a broad category of fibrous structural proteins that make up human skin, hair, and nails. Monomerically, keratin forms intermediate filaments which subsequently form these macrostructures. Keratin has the ability to self‐assemble into fibrous scaffolds.^71^ This creates an ideal matrix allowing for cellular permeation and proliferation. The wide availability of keratin makes it a desirable natural resource of biomaterials. More than 30 different cytokines and factors residual from hair morphogenesis might be useful in cardiac repair, augmenting myocyte cell viability, migration, and gene expression.^72^ Three of these factors, namely, NGF, TGF‐ β1, and BMP4, were found to support angiogenesis. Also, keratin injections showed no evidence of inflammation, and keratin‐based scaffolds were found to regenerate nerve function and foster neuromuscular recovery.^71^ Hydrogels made from keratin are notably biocompatible, have the proclivity to self‐assemble, and efficiently integrate into environments.

*Matrigel*: Matrigel is a gelatinous protein mixture, secreted by Engelbreth–Holm–Swarm (EHS) mouse sarcoma cells, and commercially available under different trade names marketed by multiple companies. It is usually used as substrate coating to improve cell adhesion; however, it can also be used as an injectable hydrogel for cardiac repair. Kofidis et al. employed matrigel for delivery of embryonic stem cells to an infarcted myocardium, where the cells were encapsulated in the matrigel injected in the infarcted left ventricle.^73^, ^74^ Their study proved that injectable matrigel may help in fixing the heart's shape, geometry, and functionality^74^ after MI. Similarly, Zhang et al. showed that a mixture of collagen, matrigel, and cells as a source of cardiomyocytes offered the same functionalities once injected in vitro, as Kofidis and his colleagues demonstrated in their later research.^75^ Furthermore, matrigel can inhibit cell death and enhance vascularization after being introduced to the infarcted region.^76^

*Decellularized ECM*: In order to better mimic the composition of the native cellular microenvironment a number of groups have investigated the use of hydrogels from decellularized tissues or extracellular matrices (ECMs). ECM hydrogels replicate the native cellular microenvironment with specific characteristics based on the tissue from which they are extracted. They can provide polymeric hydrogels with native chemical and biophysical cues and therefore improve the attachment, survival and function of the transplanted cells. Besides, each tissue has a distinct composition of fibrous proteins, proteoglycans, and glycosaminoglycans that make up its ECM.^77^ Recent studies have shown that ECM hydrogels made from decellularized cardiac tissues can help in repairing scarred myocardium after MI. Injection of ECM hydrogel from the myocardial matrix, into rats LV showed no embolization or ischemia, and no adverse effect on neighboring tissues or cardiac rhythm.^78^ Additionally, when the interaction with human blood was evaluated, the decellularized matrix had no adverse effect on activated partial thromboplastin time or prothrombin time across different blood concentrations, verifying its hemocompatibility.

#### 4.3.2 Synthetic Hydrogels

Synthetic hydrogels are of particular interest in cardiac repair and other tissue engineering applications due to their strong mechanical properties and easily controllable features,^79^ Figure 1b. Here, we discuss various synthetic hydrogels which have been used in tissue engineering or regenerative medicine and have potential for application in cardiac repair.

*Poly(acrylic acid) Derivatives*: Poly‐acrylic acid (PAA), Figure 1b(i), has several derivatives that are used as biomaterials. Poly‐2‐hydroxyethyl methacrylate, also known as poly‐HEMA, is hydrolytically stable. The cross‐linking mediator can predict its permeability and hydrophilicity. The disadvantage of PAA‐derivatives is that they are not completely biodegradable.^4^ A dextran‐modified poly‐HEMA has been developed that is found to be degradable by specific enzymes. Attaching the poly‐HEMA to oligo‐d‐lactide to form a gel phase of the poly‐HEMA without using lethal chemicals, have also been reported.^80^

Another important derivative of PAA, worth mentioning, is poly‐*N*‐isopropyl‐acrylamide, or PNIPAAm, which is commonly used in tissue engineering for its temperature‐dependent biphasic behavior. It can change its phase from liquid to gel‐phase above its lower critical solution temperature (32 °C in water). Thus, it can be injected into the body as a prepolymer solution which can turn to a gel‐like phase at body temperature after injection,^4^ which makes this polymer advantageous. The transition depends only on the body's temperature and is not based on a specific timing which makes them even more beneficial. On the other hand, these polymers are nondegradable and may be toxic, teratogenic, and/or carcinogenic.^4^ In an earlier study, a biodegradable PNIPAAm hydrogel was injected in infarct heart and was found to result in improvement of heart function.^81^ Similarly, in another report, PNIPAAm with a growth factor and antioxidants showed significant enhancement of MSC growth within the hydrogel providing a suitable microenvironment for heart cells to function.^82^ Although the synthetic poly(acrylic acid) derivatives have promising potential for application in the treatment of cardiac tissue damage, their biocompatibility and inflammatory response may arise which are needed to be resolved for their extensive application in the future.

*Polyethylene Glycol or Polyethylene Oxide and Copolymers*: Polyethylene glycol (PEG) or polyethylene oxide (PEO), Figure 1b(ii), is a biocompatible and hydrophilic polymer which is also FDA approved for several biomedical applications. PEG is specifically used to prepare biological conjugates,^83^ modify surfaces,^84^ and stimulate cell membrane synthesis.^4^ It does not cause immune responses and has low cell adhesion and protein binding.^85^, ^86^ It is produced from the ionic polymerization of ethylene oxide and has end hydroxyl groups that facilitate the formation of PEG macromers which in turn contribute to its chain polymerization. The PEG macromers can be injected in the injured sites because of their low toxicity. PEG in its gel phase is usually used as a scaffold and it is coated to inhibit interactions between encapsulated cells and the poly­mer itself.^87^, ^88^, ^89^, ^90^ The gel phase can be manufactured via UV photo‐polymerization of the polyethylene oxide with acrylate ends in the presence of R‐hydroxy acid.^91^ When interaction with proteins is needed, the hydrogel can be reinforced with bioactive peptides^3^ a property required for biomaterials used in tissue engineering and regenerative medicine.^92^

In addition, copolymers of the PEG have been found to be useful in drug delivery.^93^, ^94^ For example, PEG‐PPO‐PEG is an interesting triblock copolymer made of PEG and poly­propylene oxide (PPO) which is a thermo‐sensitive polymer that can change to a gel phase at body temperature.^95^ The drawback is that it is not biodegradable. To overcome this issue, a diblock (or triblock) of PEG and polylactic acid (PLA) was developed based on the knowledge that the PLA is biodegradable and harmless.^96^ In an earlier study, PEG was used for understanding the cardiomyocytes–matrix interactions in a 3D microenvironment. The results showed an increase in viability and functionality of encapsulated cardiomyocytes.^97^ In another study, the embryonic stem cells (ESCs)‐encapsulated PEG hydrogels demonstrated ESCs differentiation to the cardiomyocytes lineage and the cells showed cardiac‐like activity.^98^ Wang et al. injected bone marrow‐derived MSC‐encapsulated PEG‐PCL‐PEG copoly­mer mixed with cyclodextrin in the MI region of a rabbit heart and observed formation of dense vessel network at the infarct site with significantly reduced cardiac infarction.^99^ All of these studies demonstrated the superior activities of PEG copolymers in the treatment of MI.

*Polyvinyl Alcohol*: Polyvinyl alcohol, Figure 1b(iii), or PVA, is produced through the hydrolysis of polyvinyl acetate. The PVA hydrogel is developed using either physical or chemical cross‐linking. Chemical cross‐linking can potentially be harmful, so some recent studies have focused on replacing the chemical cross‐linking with photocrosslinking. The solubility and hydrophilicity of manufactured hydrogel depend on the molecular weight and the degree of hydrolysis. PVA can connect to multiple biological molecules.^3^, ^4^ It can be used as a matrix due to its elasticity and role in improving the diffusion of mechanical signals. PVA is a neutral hydrogel and consequently its adhesion properties are relatively minimal but can be enhanced by mixing it with biological factors. PVA hydrogel has strong mechanical properties and a low rubbing coefficient.^3^ All these excellent properties of PVA exhibit promising tissue engineering applications, especially cardiac tissue repair.

*Polyphosphazene*: Polyphosphazene, Figure 1b(iv), has attracted scientists' interest because of its biodegradability. By modifying its side‐chain organization the dynamics behind its degradation process can be regulated. The polyphosphazene polymer includes interchanging atoms of phosphorus and nitrogen and two side groups connected to each phosphorus atom. A transitional product, poly‐dichlorophosphazene, is formed before creating the final polymer. The gel phase of the end product, polyphosphazene, is a result of its hydrophilic backbone and its flexibility acquired following different substitution reactions. It can only be modified to create a thermo‐responsive hydrogel. From the polyphosphazenes, nonionic and ionic hydrogels can be prepared. The latter reacts with variation in pH or ionic strength and consequently they might be useful for protein drug delivery.^4^

*Polypeptides*: Peptides, Figure 1b(v) can form nanofiber hydrogels by means of charge interactions, hydrogen bonding, and other interactions.^100^ The use of peptides is very beneficial since they can act as a scaffold that mimics a natural extracellular matrix (ECM).^101^ Different types of peptide hydrogels can be formed. Thermo‐responsive peptide hydrogels have been made of elastin‐like proteins that contain tropoelastin.^102^, ^103^ The ion‐induced cross‐linked peptide hydrogels change to a gel‐like structure based on interactions with a concentration of salt, which will increase the ion strength, specifically by minimizing the repulsion occurring in the positively charged side‐chain.^104^ Some peptides can form pH‐responsive hydrogels. Two amino acids are essentially responsible for this activity: valine and lysine. In liquid phase, hydrophobic valine and hydrophilic lysine are on opposite sides of the peptide's secondary structure and this combination creates hydrogen bonds within the structure. As the pH decreases, the valine and adjacent lysine repel each other. As a result, the peptide structure unfolds and the hydrogel dissolves. Some peptides upon being attached to the fluorenyl‐methoxy‐carbonyl can form a gel‐like hydrogel with enhanced cell adhesion and proliferation properties.^79^

#### 4.3.3 Composite Hydrogels

In addition to various natural and synthetic polymer‐based hydrogels, a number of hybrid and composite hydrogels have also been tried for application in cardiac repair. Some specific examples include ECM‐fibrin hydrogels,^105^ alginate‐chitosan hydrogels,^106^ and ECM‐polyethylene glycol hydrogels.^107^ Due to the benefits of both fibrin and alginate as biomaterials for injectable hydrogels, a composite of these materials was used for cardiac repair after MI. Results showed that the expansion of the infarcted region of the left ventricle stopped upon injection of the hydrogel. Additionally, myocardial stiffness was relatively higher than those of control groups and fibrous collagen in the myocardium border region was increased.^108^ Hence, the soluble collagen content in the infarcted zone was decreased, which accounts for the deceleration of damaged tissue enlargement. Thus, injection of fibrin‐alginate as well as other suitable composite hydrogels may provide an effective treatment to suppress the expansion of infarcted tissue.

**Figure 2:**
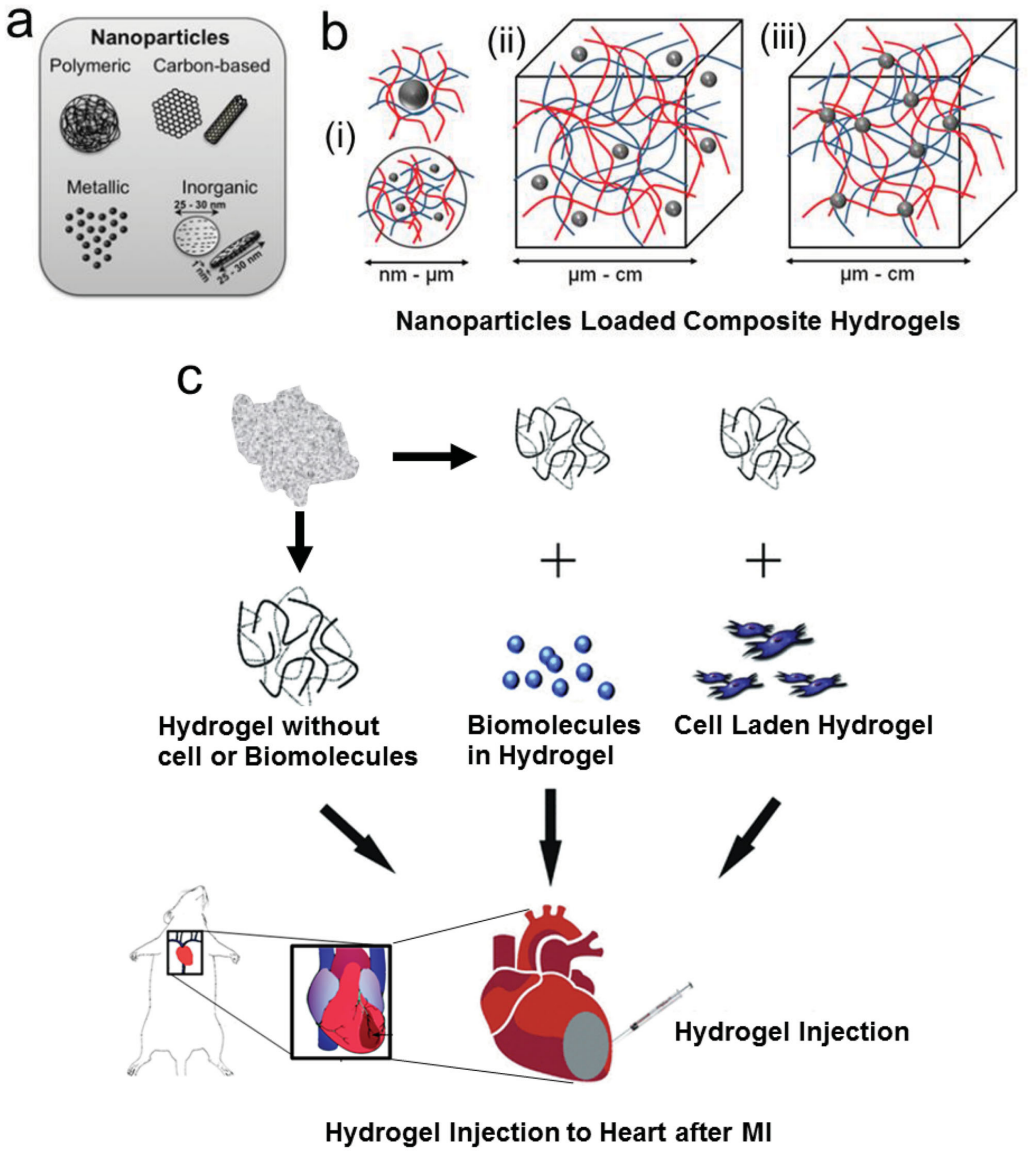
Schematic representation of nanoparticle loaded injectable composite hydrogels, and delivery of various hydrogels including acellular hydrogels alone, acellular hydrogels with various biomolecules and hydrogels with various cells. a) A range of nanoparticles such as polymeric nanoparticles, metallic‐metal oxide based nanoparticles, inorganic nanoparticles, and carbon‐based nanomaterials can be incorporated in hydrogels to make nanocomposite hydrogels. Adapted with permission.^109^ b) The common strategies for incorporation of nanoparticles in hydrogels: i) stabilization of inorganic or polymeric nanoparticles by nano–micro‐sized hydrogel particles, ii) noncovalently immobilized nanoparticles in a hydrogel matrix, and iii) covalently immobilized nanoparticles in hydrogel matrix. Adapted with permission.^110^ c) Different strategies of injectable hydrogel delivery for treatment of MI. The injectable hydrogels can be delivered alone without any biomolecules or cells, with some biomolecules as carriers or with cells as a 3D matrix. Adapted with permission.^76^ Copyright 2010, Royal Society of Chemistry.

A recent strategy for achieving multiple functionalities in hydrogels is to incorporate various nanoparticles in them, thereby developing nanocomposite hydrogels. The nanoparticles to be incorporated can be polymeric, metallic, ceramic, inorganic, or carbon‐based as shown in **Figure** **2**a^109^ while the strategies of incorporation can be physical entrapment, noncovalent immobilization, or covalent immobilization as shown in Figure 2b.^110^ The different strategies for the delivery of injectable composite hydrogels as well as other injectable hydrogels including acellular hydrogel alone, acellular hydrogel with some biomolecules, and the cell‐laden hydrogel delivery are shown in Figure 2c.^76^ Recent advances in the applications of these hydrogels in cardiac repair and regeneration with specific examples are discussed in the section below.

## 5 Recent Advances in Application of Injectable Hydrogels in Cardiac Tissue Repair

While numerous hydrogels have been synthesized for tissue engineering, only a few of them have been tested for cardiac applications. However, some of these hydrogels have shown strong promises in enhancing the repair of infarcted myocardium. Acellular injectable hydrogels without any therapeutic biomolecules were found to thicken the myocardial wall, thereby reducing abnormal stresses when injected directly after an infarction.^7^, ^13^ Other reported improvements were enhanced cardiac function, reduced infarct size, and induced neovascularization, using injectable hydrogels as carriers for various biomolecules^111^ and as carriers of cells.^13^, ^77^, ^112^, ^113^, ^114^ In subsequent sections, we discuss the recent advances in applications of injectable hydrogels in cardiac tissue repair.

### 5.1 Acellular Hydrogels for Treatment of MI

**Figure 3:**
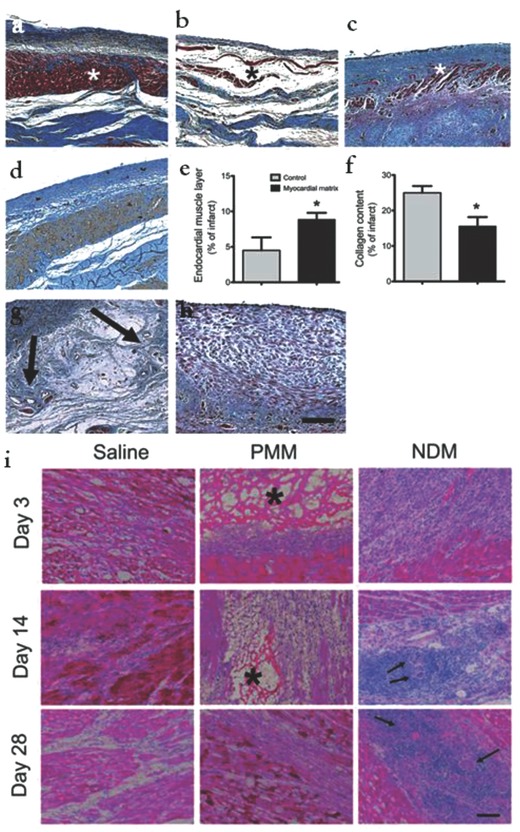
a–h) Enhancement of cardiac muscle and reduction of infarct fibrosis using myocardial matrix. Histological characterization of infarcted pig hearts: Masson's trichrome staining images are representative of six matrix‐injected pigs and four control animals. a) Matrix‐injected hearts had a distinct, thick endocardium (red stained muscle, indicated with an asterisk). b) Noninjected control animals had a loose fibrillar layer (blue) beneath the endothelium. c) In saline injected control animals, the endocardium was moderately thickened with minimal muscle (red). d) An adjacent tissue section for the matrix‐injected animal in a) stained for cardiac troponin‐T, indicating the presence of cardiomyocytes. e) Area of endocardial layer of muscle as a proportion of the infarct. f) Percentage of collagen content in the infarcts. Data are means ± SEM and were obtained from Masson's trichrome slides a–c). *P < 0.05 (Student's t test). g,h) Matrix‐injected hearts contained foci of neovascularization in the area below the endocardium (g, arrows), but none of the saline or noninjected control hearts showed these areas of neovascularization h). Scale bar, 200 mm. i) Myocardial matrix is biocompatible and biodegradable. Representative histological sections of cell infiltration in matrix‐injected rat hearts injected with saline, PMM, or NDM at days 3, 14, and 28. Inflammation and multinucleated giant cells are present in the NDM groups at days 14 and 28 (arrows). The PMM (asterisk marked porous network) was completely degraded by 28 d. Scale bar, 200 mm. Reproduced with permission.^78^ Copyright 2013, American Association for the Advancement of Science.

As mentioned earlier, acellular hydrogels have exhibited promising results in cardiac repair as both a bulking agent to provide mechanical support to the infarcted heart when injected alone, and as a carrier for various biomolecules including growth factors, cytokines, and DNA plasmids. Several scientists have also focused on acellular biomimetic hydrogels without biomolecules in order to focus on materials that give the proper biological and chemical cues that mimic the native microenvironment. For example, in a recent study, an injectable ECM hydrogel derived from decellularized porcine myocardial tissue was used to reverse the negative remodeling process in infarcted myocardial tissue.^78^ Upon injecting the hydrogel into infarcted pig hearts, the tissue self‐assembled into a porous scaffold, allowing cell infiltration. Histological characterization showed that the hearts that were injected with the ECM hydrogel developed a distinct layer of endocardium, while the control group exhibited a fibrillary layer and the saline‐injected control endocardium was moderately thickened, **Figure** **3**a–c. ECM hydrogel‐treated myocardial tissue exhibited 10% less collagen content compared to control groups, as well as the presence of cardiomyocytes as evidenced by cardiac troponin‐T staining, Figure 3d. This reduction in fibrosis is essential for heart repair since fibrous tissue is noncontractile, which inhibits the heart's ability to pump blood properly. The tissue injected with the ECM hydrogel showed foci of neovascularization as well, whereas the control groups did not, Figure 3g,h. This observation provided evidence of cardiac regeneration due to the fact that blood flow to the infarct zone provides oxygen essential for cardiomyocytes to grow.

Rodents were used to assess the biocompatibility and degradation properties of the myocardial ECM hydrogel, Figure 3i. The ECM hydrogel exhibited complete degradation by 28 d postoperation. The group receiving nondecellularized porcine myocardial matrix (NDM) also showed similar degradation in addition to an immune response exhibited by inflammation and multinucleated giant cells. This further confirms that the ECM itself does not elicit an immune response, as long as the matrix is successfully decellularized. These positive results provide a base of support to move this ECM hydrogel toward clinical studies. The detailed study elicits how the components of ECM can be leveraged in engineering cardiac tissue.

### 5.2 Injectable Cell‐Laden Hydrogels for Cardiac Repair

**Figure 4:**
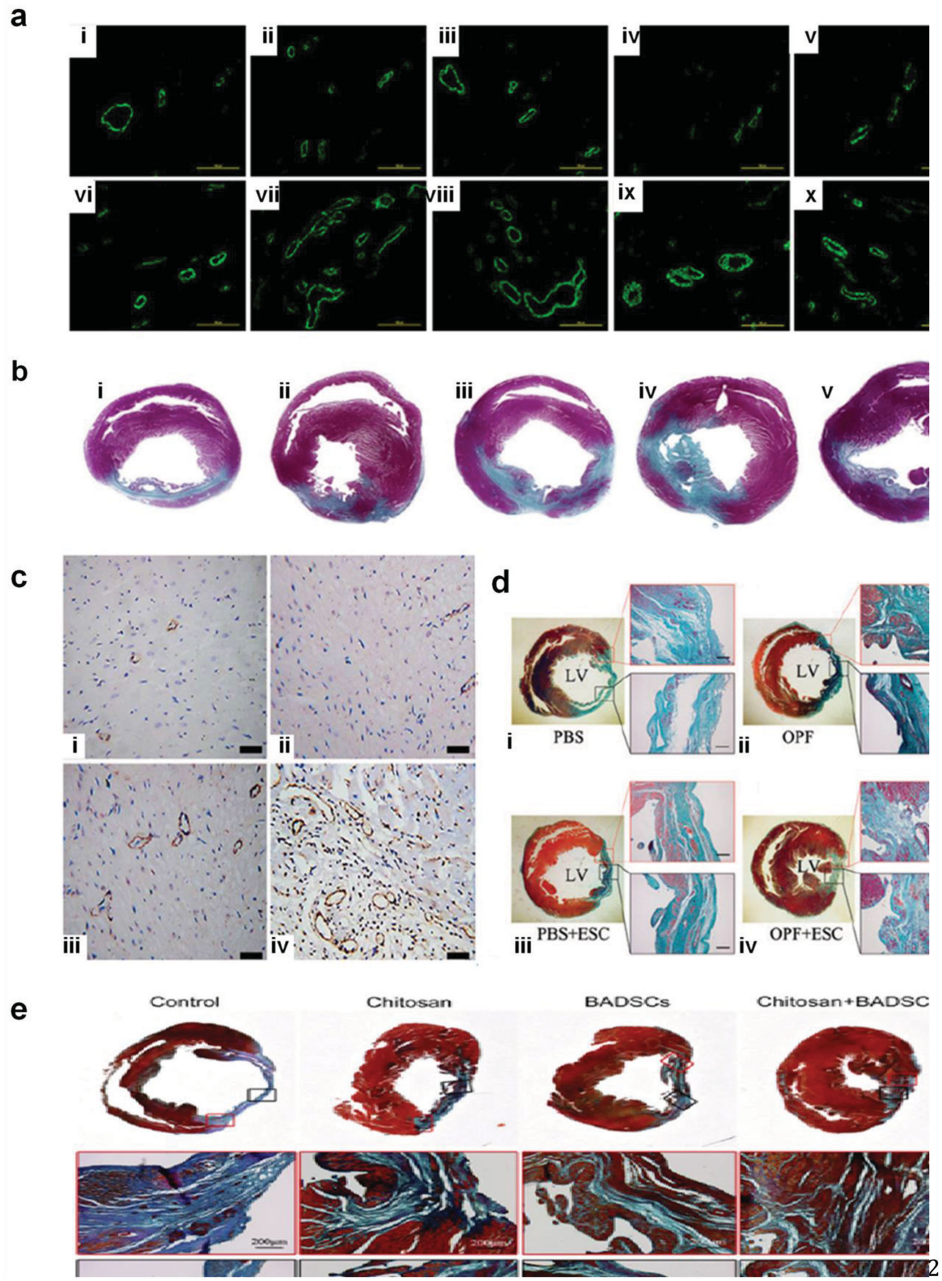
Arterial staining and histological morphology of excised rat hearts treated with injected hydrogels 4 weeks post MI. Image (a) shows Alpha‐SMA positive vessels in the infarct and peri‐infarct zones, 4 weeks after MI. Panels (i) and (ii) show control (saline injection) peri‐infarct and infarct zone respectively, (iii) and (iv) show peri‐infarct and infarct treated with PF1%, (v) and (vi) show those treated with PF2% respectively, (vii) and (viii) treated with TF1%, respectively, and (ix) and (x) treated with TF2%, respectively. In image (b) 4 weeks after the MI the treated rat hearts were cut into equal transverse slices to show the gross appearance and histological morphology (i) in that of the untreated saline control group where saline injection was only used. The PF1% hydrogel injection in (ii), the PF2% hydrogel injection in (iii), the TF1% hydrogel injection in (iv), and the TF2% hydrogel injection in (v). Reproduced with permission.^13^ Copyright 2014, Elsevier. Image (c) shows immune‐histochemical staining for the smooth muscles for the four groups 30 d after MI. Panel (i) shows the control group, (ii) shows hydrogel group, (iii) shows BMMNC group, and (iv) shows BMMNC + hydrogel group. Reproduced with permission.^124^ Image (d) shows the staining with Masson trichrome of the infarcted wall forcollagen (green) and muscle (red) of the four groups. (i) PBS‐only group of the four groups; (ii) OPF‐only group; (iii) PBS + ESC group; and (iv) OPF + ESC group. Reproduced with permission.^19^ Copyright 2014, Elsevier. Image (e) also shows staining with Masson's trichrome of the infarct area and shows similar level of tissue improvement with BADSCs treatment alone or Chitosan alone compared to the control group with Chitosan hydrogel delivery of BADSCs resulting in the greatest healing and infarct reduction. Reproduced with permission.^124^

Various injectable hydrogels, including tetronic‐fibrinogen (TF) and PEG‐fibrinogen (PF) conjugate hydrogels, have been used to enhance the efficacy of stem cell delivery in infarcted myocardium,^13^, ^112^, ^113^, ^114^ **Figure** **4**a,b. In one study, a left thoracotomy was performed by antero‐lateral incision, followed by the injection of hydrogel precursor solution on four sides of the infarcted region.^13^, ^77^ UV light was used to catalyze the crosslinking process.^13^ Results showed that hydrogel treatment to the left ventricle (LV) led to an increase in wall thickness, which in turn increased the survival of viable cardiac tissue where infarction took place.^115^, ^116^ Remodeling of the LV was revealed to some extent via echocardiography. The prevention of LV dilation and fractional shortening deterioration were observed in addition to minimal wall thinning.^23^ Both cross‐linked PF and TE hydrogels with BMNCs showed increased neovascularization, leading to improvements in cardiac function. Injecting TF hydrogel and saline led to the same arteriole density, while PF 2% exceled the former by offering a greater restoration of heart function and neovascularization.^13^, ^117^

In another study, bone marrow derived stem cells (BMSCs) from rabbit tibia were encapsulated in Dex‐PCL‐HEMA/PNIPAAm hydrogels and injected into their infarcted myocardium after performing left thoracotomy and ligating the left coronary artery. One month later, the percent infarcted size was obtained from the ratio of infarcted wall to all the surface area of left ventricle (LV).^77^, ^118^ The BMMNCs survived up to 3 d in hydrogel. Notably, the cell density in the animal group injected with cell‐laden hydrogel was much higher compared to that in the groups with either cells or hydrogels alone. After 48 h there was no significant difference in the results between the four groups on the level of LV end‐systolic dia­meter (LVESD), LV end diastolic diameter (LVEDD), and LV ejection fraction (LVEF). However, the echocardiography results after 30 d demonstrated that LVEF of the group that received BMMNCs encapsulated in the hydrogel was more significant than the other groups. In contrast, this group had lower levels in LVEDD and LVESD. Thus, the injected hydrogels enriched with encapsulated cells increased cell engraftment, where hydrogel could serve as the ECM, leading to an improved rate of cell retention and survival in the infarcted zone.[[qv: 77,119,120]]

In a third study, embryonic stem cells (ESCs) extracted from mice were encapsulated into an oligo [poly(ethylene glycol) furmarate] (OPF) hydrogel and injected at the border of the infarcted region 1 week after inducing MI in mouse hearts. After 24 h, the hearts were excised, frozen and bisected into five different sections, followed by tracing Green Fluorescent Protein (GFP)‐labeled mouse ESCs via fluorescent microscope. After 4 weeks, immunochemical staining was used to determine graft size in the specimens.^19^

The synergistic effect of OPF hydrogel and ESCs was evident by the fact that ESCs in OFP differentiated into cardiomyocytes, ESC count was higher, the ESCs were aggregated in the hydrogel, LV function for mice with ESC and OPF showed the most improvement, infarction size diminished the most, collagen deposition was minimal, no signs of prolonged inflammatory response were observed, as well as a significant increase in neovascularization was exhibited. Also, the OPF hydrogel completely degraded.^19^, ^121^, ^122^, ^123^

In a recent study, cardiac progenitor cells (CPC) were found to exist in the form of a CD29 positive population.^124^ Chitosan hydrogel was tested with incorporation of these cells as a method to repair damaged myocardium. CD29+ brown adipose derived stem cells (BADSCs) were obtained from rats, and then rats were subdivided into four groups containing 20 rats each. Each group received one of the following treatments: PBS only, chitosan hydrogel, BADSCs only, and BADSCs encapsulated in chitosan hydrogel. These treatment groups were put into place after the left coronary artery was ligated.^124^ Lateral investigations were conducted to identify the cardiac differentiation of BADSCs in vitro, where they were treated with and without chitosan, Figure 4e. As a result, the treated groups showed significant favorable results. In detail, the cross‐linked strained myofilaments were better organized than the control group. Analysis showed significant increase in the levels of collagen 1 when using chitosan treated with BADSCs compared to the control group. In addition, applying chitosan hydrogel led to enhancement in cell retention in the infarcted site, leading to survival of the grafted cell in ischemic myocardium.^124^

Furthermore, chitosan + BADSCs had a synergistic effect as they led to a significant recovery of cardiac function—significant reduction took place in the left ventricle infracted zone, which led to a marked increase in the number of cells responsible for cardiomyocytes differentiation, as well as neovascular formation.^124^

These studies clearly demonstrate how encapsulating cells in hydrogels can increase their therapeutic efficacy in terms of positive cardiac remodeling. The material properties of the hydrogel can be tailored specifically to provide the cell source with the correct chemical, mechanical, and biological signals to differentiate into the desired lineage, such as cardiomyocytes. Furthermore, hydrogels provide the cells with a mechanically stable, biocompatible environment to protect the cells from being washed away by the body's defense mechanisms. Therefore, these tunable properties give hydrogels great potential to be an integral part of the success of cell therapy in treating myocardial infarction.

### 5.3 Carbon Nanotube‐Embedded GelMA Hydrogels for Cardiac Constructs

**Figure 5:**
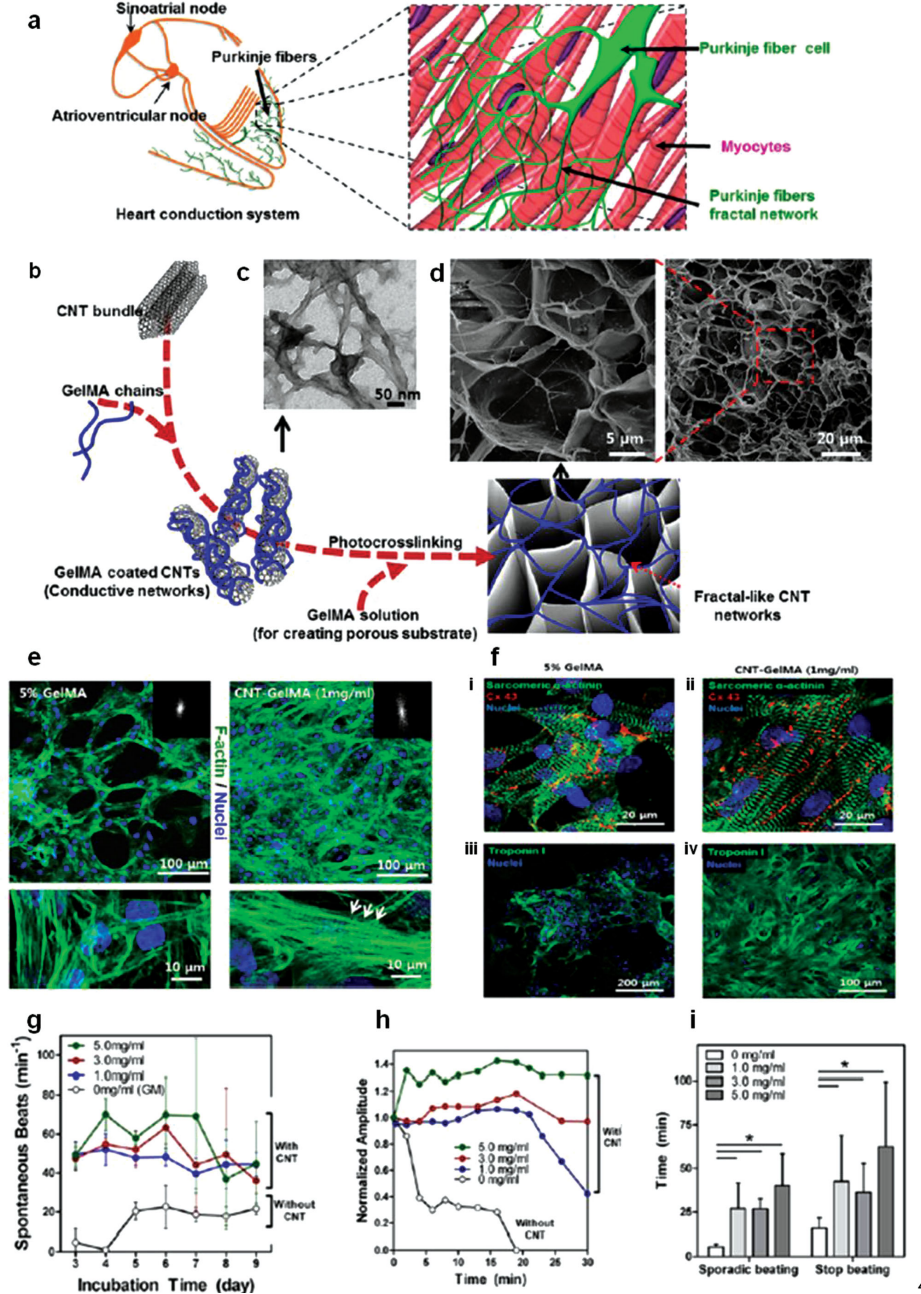
a–d) Structural, physical, and electrical properties of CNT‐GelMA hydrogels: a) schematic diagram illustrating the isolated heart conduction systems showing the purkinje fibers, which are located in the inner ventricular walls of the heart. b) Preparation procedure of fractal‐like CNT networks embedded in GelMA hydrogel. c) TEM image of GelMA‐coated CNTs. d) SEM images show porous surfaces of a 1 mg mL^−1^ CNT‐GelMA thin film. e,f) Adhesion, maturation, alignment, and phenotype of cardiac cells on CNT‐GelMA hydrogels. e) Confocal images of cardiomyocytes after culturing for 5 d on pristine GelMA and 1 mg mL^−1^ CNT‐GelMA revealed more uniform cell distribution and partial cell alignment on CNT‐GelMA. Higher magnification images showed well‐elongated cardiac cells and well‐developed F‐actin cross‐striations (bottom right, white arrows) on CNT‐GelMA but not on pristine GelMA (bottom left). f) Immunostaining of sarcomeric R‐actinin (green), nuclei (blue), and Cx‐43 (red) revealed that cardiac tissues (8 d culture) on (i) pristine GelMA and (ii) CNT GelMA were phenotypically different. Partial uniaxial sarcomere alignment and interconnected sarcomeric structure with robust intercellular junctions were observed on CNT‐GelMA. Immunostaining of Troponin I (green) and nuclei (blue) showed much less and more aggregated Troponin I presence on (iii) pristine GelMA than on (iv) CNT‐GelMA. g–i) Improved mechanical integrity and advanced electrophysiological functions of cardiac tissues on CNT‐GelMA. g) Spontaneous beating rates of cardiac tissues recorded from day 3 to day 9 on a daily basis. h) CNTs protected cardiac tissues against damages by heptanol. Plots of spontaneous beating amplitude over time (5 d culture) for 0–5 mg mL^−1^ CNTs in GelMA in response to 4 × 10^−3^ m heptanol. i) Time lapse before sporadic beating and stop of beating induced by heptanol (*p < 0.05).Reproduced with permission.^125^ Copyright 2013, American Chemical Society.

In the case of engineering heart tissue, scaffolds do not usually exhibit the capability to conduct electrical current. However, the native tissue contains purkinje fibers that do in fact conduct electricity, allowing the heart to beat. A recent study employed carbon nanotubes (CNT) embedded GelMA hydrogel with improved mechanical and electrical properties to enhance the differentiation of the mesenchymal stem cells (MSCs) into cardiac cells, **Figure** **5**. Another recent study showed a promising approach for high‐performance cardiac scaffold materials that can be created with the incorporation of CNTs into a photo‐cross‐linkable GelMA hydrogel.^125^ The cardiac cells in CNT‐GelMA were observed to be in an elongated state and F‐actin fibers were demonstrated to be intact and more homogeneous in comparison with pristine GelMA. Immunostaining showed a significant increase in both sarcomeric α‐actinin and Troponin I on the CNT‐GelMA in comparison with the pristine GelMA. Also, sarcomere alignment, sarcomere interconnected structures, and troponin aggregation were observed on the CNT‐GelMA. Both CNT‐GelMA and pristine GelMA can generate synchronous beating activity. However, after the spontaneous beating rates were recorded over a 6 d period, the CNT‐GelMA showed a beating rate average that was both more stable and three times greater than the pristine GelMA. In order to study the cardiac beating, two model drugs known for disrupting the biological processes necessary for continued heart beats were introduced: heptanol and doxorubicin. In the case of heptanol, the gap‐junctional beating propagation is inhibited in the heart. However, in the CNT‐GelMA, synchronous beating was observed even after the drug was administered. In fact, it took 40–65 min for synchronous beating to stop in the CNT‐GelMA, as opposed to only 20 min in the pristine GelMA. This demonstrated that the CNT in the hydrogel assisted in maintaining the pathway that the heptanol disables and enhanced beating amplitudes and rates.^125^

### 5.4 Injectable Hydrogel‐Based Gene Delivery Systems

**Figure 6:**
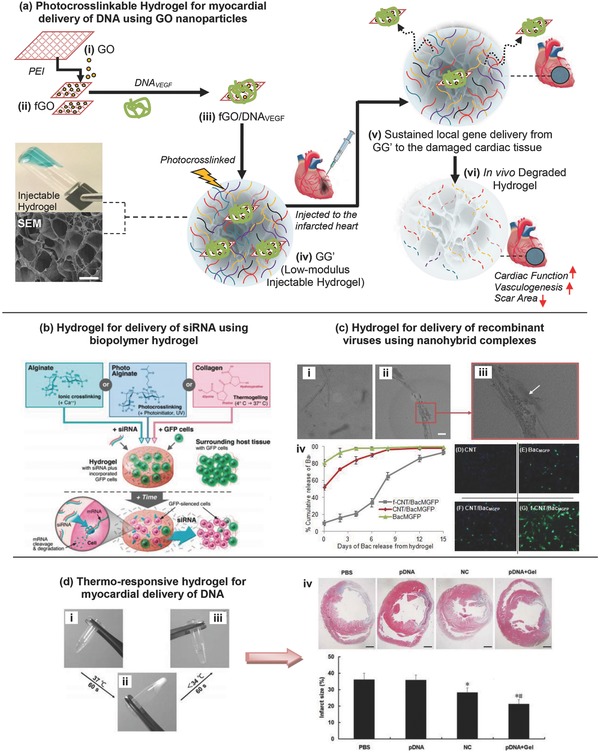
Injectable hydrogels for gene delivery applications. a) Photocrosslinkable hydrogel for myocardial delivery of vascular endothelial growth factor (VEGF) carrying gene using cationic functionalized graphene oxide (fGO) nanoparticles. Schematic of stepwise formulation process for direct intramyocardial injection of damaged heart with acute myocardial infarction. Reproduced with permission.^126^ Copyright 2014, American Chemical Society. b) Delivery of siRNA using biopolymer hydrogel—schematic of hydrogel formation for delivery of siRNA and subsequent inhibition of gene expression in incorporated and neighboring cells. Biomaterial solutions of alginate, photo alginate, or collagen are mixed with siRNA and GFP‐positive cells, and hydrogels are then formed by crosslinking, photo‐crosslinking, or thermos‐gelling, respectively. The siRNA diffuses through the hydrogel to affect incorporated cells, and it is also released from the hydrogel to locally affect surrounding cells that are part of the host tissue. Reproduced with permission.^127^ Copyright 2009, American Chemical Society. c) Hydrogel for delivery of recombinant viruses using nanohybrid complexes. Controlled release of baculovirus (Bac) using CNT reinforced hydrogel. TEM images of (i) nonfunctionalized CNT with Bac, (ii) CNT functionalized Bac with arrow showing the baculovirus bound to CNT surface in magnified image (iii). Scale bar indicates 100 nm length. Self‐assembled nanocomplex of cationic PAA functionalized CNTs (f‐CNT) were hybridized with anionic Bac. (iii) Cumulative release kinetics of baculovirus from denatured collagen hydrogel (2.5 mg mL^−1^) impregnated with Bac–CNT nanocomplex (v–viii–G) rMSCs were overlaid on the collagen hydrogel formulated with CNT (25 μg mL^−1^), BacMGFP, CNT/BacMGFP or functionalized CNT f‐CNT/BacMGFP. Abbreviations: TEM = transmission electron microscope; CNT = carbon nanotube; rMSCs = rat mesenchymal stem cells; PAA = poly (acrylic acid), MGFP = Monster Green Fluorescent Protein. Reproduced with permission.^128^ Copyright 2014, Elsevier. d) Thermo‐responsive hydrogel for myocardial delivery of plasmid DNA. Thermosensitive sol‐to‐gel transition properties of biodegradable dextran‐poly(e‐caprolactone)‐2‐hydroxylethylmethacrylate‐poly(*N*‐isopropylacryl amide) (Dex‐PCL‐HEMA/PNIPAAm) hydrogel at 1.5 wt% concentration: (i) gel solution in fluidity at room temperature, (ii) the gel solution turned into gel at 37 °C, and (iii) sol–gel reversibility of gel solution at room temperature. (iv) Representative pictures of left ventricles from each group after Masson's Trichrome staining 30 d after treatments. Scale bar = 1 mm. Below images are the infarct size as percentages at 30 d. Infarct size is calculated as the ratio of infarcted to noninfarcted area of the left ventricle. Reproduced with permission.^129^

Gene therapy offers a unique opportunity to promote local healing of damaged myocardial tissue mainly by promoting new blood vessel formation and attenuating fibrosis. Some methods that are frequently used in delivering specific therapeutic genes using injectable hydrogels include upregulating or knocking down specific host genes or simply by overexpressing certain genes of therapeutic interest. One recent study aimed to improve the biofunctionality of injectable hydrogels by incorporating a gene delivery system using polyethylenimine (PEI) functionalized graphene oxide nanosheets (fGO) complexed with vascular endothelial growth factor‐165 (VEGF), all incorporated within methacrylated gelatin (GelMA), **Figure** **6**a.^126^ The addition of the fGO increased mechanical strength, reinforced the physical network of the composite hydrogels, and established strong binding of fGO to plasmid DNA for eventual transfection to the host tissue. Biocompatibility of the formulated hydrogel, evaluated using quantitative PCR and ELISA analysis of proinflammatory tumor necrosis factor α, was confirmed as the GO‐GelMA nanocomplex did not induce any cytotoxic and inflammatory effects.

Another study used hydrogels to overcome the drawbacks associated with the use of short interfering RNA (siRNA) to control gene expression, Figure 6b.^127^ The mechanism behind siRNA‐based therapy is that the mRNA that is complementary to the siRNA is degraded and therefore after transcription, the gene expression process is inhibited as needed. This study used calcium cross‐linked alginate, photo‐cross‐linked alginate, and collagen biodegradable hydrogels to encapsulate both the cells and the siRNA responsible for knocking down their gene of interest, GFP. The siRNA present in the hydrogel had the ability to affect the incorporated cells as well as locally affect the surrounding cells in the host tissue. The degradation rates of the polymers chosen in this study greatly affected the results in silencing the gene of interest. Due to the fact that the alginate degrades quickly, this study confirms that by using the degradation rates of different polymers, a sustained local release of siRNA can be delivered. When using these techniques, the parameters of the specific application need to be taken into consideration to determine the most beneficial timeline of delivery.

Viral gene delivery systems have also been shown to be effective for myocardial regeneration therapy, Figure 6c. In this case, hydrogels were used to deliver CNT hybridized baculoviruses to rat bone marrow stem cells.^128^ These hydrogels were made up of a denatured collagen matrix containing MGFP gene carrying baculoviruses wrapped in single‐walled carbon nanotubes. The hydrogel was shown to have greater mechanical properties and a more sustained delivery of the recombinant baculovirus to the cells. The group containing the functionalized gel showed a much greater number of MGPF expressing cells compared to the control group, the baculovirus group and the nonfunctionalized hydrogel group using fluorescent microscopy.

Last, in a recent study, short‐hairpin RNA (shRNA) of angiotensin converting enzyme was injected into rat myocardium postmyocardial infarction using a dextran‐poly(e‐caprolactone)‐2‐hydroxylethylmethacrylate‐poly(*N*‐isopropylacrylamide) (Dex‐PCL‐HEMA/PNIPAAm) hydrogel, Figure 6d.^129^ This study used the RNA interference gene‐technique in order to silence the gene responsible for upregulation of angiotensin converting enzyme (ACE). This is important in cardiac repair and remodeling because upregulation of ACE leads to cell apoptosis and increasing infarct size, both of which ultimately lead to heart failure. This study proved that the hydrogel sustained an extended gene expression of the shRNA responsible for the silencing gene‐technique in vivo. Thirty days after intramyocardial injection of the ACE‐shRNA plasmid‐loaded hydrogel they observed decreased ACE expression, inhibited cell apoptosis, reduced infarct size, and improved cardiac function. These results were better than either the hydrogel or the ACE‐shRNA by itself.

## 6 Conclusions and Future Perspectives

Injectable hydrogels offer huge potential for application in repair and regeneration of infarcted heart after MI. They can be used as carriers to deliver therapeutic drugs, biomolecules or cells to invoke a specific response in the infarcted heart tissues, or to form functional cardiac tissue constructs for replacement of infarcted cardiac tissues. In recent years, numerous hydrogels have been investigated for their application in cardiac repair and regeneration. Several of these hydrogels have shown great promises toward achieving cardiac tissue repair. To date, no hydrogel composition has shown both the necessary biological and mechanical properties for sufficient transition to clinical cardiac tissue repair. While naturally derived hydrogels from collagen, hyaluronic acid, chitosan, and ECM hydrogels have shown promise in the left ventricle modeling of the myocardium, synthetic hydrogels have been shown to offer better control over their properties such as degradation time, gelation time, and most importantly the mechanical stiffness of the hydrogel.

One school of thought is that hydrogels can induce stem cell homing. However, not much is known about the exchange of signals that take part in the movement of stem cells to an injured myocardium posthydrogel treatment. The importance of hydrogels also manifests itself in their ability to deliver drugs and chemical signals throughout the body to the heart. In such cases, targeted delivery and long‐term controlled sustained release are the advantages. Cytokines, such as the stem‐cell factor (SCF), granulocyte SCF, or stromal‐cell‐derived factor (SDF), may enhance cell transplantation and infiltration.^130^ When SCF was combined with G‐SCF, a 250‐fold increase in the number of circulating cells was made apparent in a model version. Cell microenvironments regulate and repair cellular fate and function. The ECM is a crucial element of the cell microenvironment that includes different chemical and physical cues.^131^ Encapsulating cells in a suitable hydrogel before transplanting or injection also increases the chances for their survival, as it increases the cell‐ECM interactions. This is why much attention has now been focused on hydrogels obtained from ECM molecules. These biomaterials are now made to replicate ECM interactions, thus providing the best way to ensure the sustainability, survival and full function of the transplanted cells.^132^

Besides, the hydrogels can be specifically designed to help in myogenic differentiation.^133^ Despite a considerable amount of research having been done on repair of infarcted heart tissue using injectable hydrogels in small animals such as rats, mice, and rabbits, research on application of hydrogel therapies on large primates and humans is still lacking. Thus, more investigations are required before the injectable hydrogel therapies with cells or biomolecules for cardiac repair are implemented in humans. Challenges in this endeavor include ensuring the survival and integration of the delivered cells in the cardiac environment and their differentiation into the required myogenic phenotypes so that they can start performing like beating cardiac cells in minimal time from the time of injection. Another challenge is the use of chemical cross‐linking which can often be harmful for the cells. Photocrosslinking, ionic cross‐linking, or temperature or pH‐based crosslinking might be alternate options; however, application of these methods in situ is often difficult. Deficiency of needed cells, the lack of full integration with the host tissue, and the absence of electrical communication through gap junctions are also among challenges that can compromise the success of the injectable hydrogel‐based stem cell therapy or cardiac repair and require further research. Research has shown improvements in wall thickness, LV repair, and vascularization of the ischemic region with the use of injectable hydrogel based therapies. Studies performed so far in rodents and pigs give hope to the potential of hydrogel based therapies for cardiac repair in primates and humans.
